# Causes and predictors of early readmission after percutaneous coronary intervention among patients discharged on oral anticoagulant therapy

**DOI:** 10.1371/journal.pone.0205457

**Published:** 2018-10-31

**Authors:** Mahesh K. Vidula, Cian P. McCarthy, Neel M. Butala, Kevin F. Kennedy, Jason H. Wasfy, Robert W. Yeh, Eric A. Secemsky

**Affiliations:** 1 Division of Cardiovascular Medicine, Department of Medicine, Hospital of the University of Pennsylvania, Philadelphia, Pennsylvania, United States of America; 2 Department of Medicine, Massachusetts General Hospital, Boston, Massachusetts, United States of America; 3 Division of Cardiology, Department of Medicine, Massachusetts General Hospital, Boston, Massachusetts, United States of America; 4 Saint Luke's Mid America Heart Institute/University of Missouri–Kansas City, Kansas City, Missouri, United States of America; 5 Smith Center for Outcomes Research in Cardiology, Department of Medicine, Beth Israel Deaconess Medical Center, Boston, Massachusetts, United States of America; University of Florida, UNITED STATES

## Abstract

Patients discharged on oral anticoagulant (OAC) therapy after percutaneous coronary intervention (PCI) represent a complex population and are at higher risk of early readmission. The reasons and predictors of early readmission in this group have not been well characterized. We identified patients in an integrated health care system who underwent PCI between 2009 and 2014 and were readmitted within 30 days within this health care system. Of the 9,357 patients surviving to discharge after the index PCI, 692 were readmitted within 30 days (7.4%). At the time of readmission, 143 had been discharged from the index PCI hospitalization on OACs (96.5% on warfarin) and 549 had not been discharged on OACs, with readmission rates of 12.9% and 6.7%, respectively (p<0.01). The most common reason for readmission among all patients was chest pain syndromes (21.7% on OACs, 34.4% not on OACs). However, bleeding represented the next most frequent cause of readmission among patients on OACs (14.0% on OACs vs 6.0% not on OACs, p<0.01). Among patients on OAC therapy, peripheral arterial disease (odds ratio [OR] 1.66, 95% confidence interval [CI] 1.07–2.57, p = 0.02) and nonelective PCI (OR 1.91, 95% CI 1.17–3.12, p<0.01) were found to be independent predictors of 30-day readmission. During rehospitalization, compared to patients not on OACs, patients on OACs suffered a higher unadjusted rate of mortality (6.3% vs 1.8%, p<0.01) and a longer length of stay (6.4 ± 7.1 days vs 4.9 ± 6.8 days, p = 0.02). In conclusion, patients discharged on OAC therapy after PCI are commonly readmitted, with bleeding representing a major reason. These readmissions are associated with high mortality and longer lengths of stay. Interventions targeted towards optimizing discharge planning for these complex patients are needed to potentially reduce readmissions.

## Introduction

Readmissions following percutaneous coronary intervention (PCI) are expensive and burdensome for patients. A recent review found that rates of 30-day readmission after PCI range from 4.7–15.6%, and readmitted patients may be at increased risk of death at 1 year [1]. The 30-day readmission rate is also used as a quality metric for hospitals, since readmissions may reflect the quality of care the patient received at the time of index hospitalization or after discharge [2]. The Centers for Medicare & Medicaid Services (CMS) Hospital Readmissions Reduction Program now penalizes hospitals for higher than expected 30-day readmission rates for certain medical conditions, including acute myocardial infarction (MI) and heart failure [3]. Since many of these patients undergo PCI during their hospitalization, reducing readmissions after PCI has become a priority for hospital systems, and interventions targeted towards reducing readmissions post-PCI have been implemented [2,4]. In addition, the CMS recently announced a new voluntary bundled payment model, the Bundled Payments for Care Improvement Advanced, which ties reimbursement for PCI to several quality measures including readmission [5].

Causes and predictors of readmission after PCI have previously been explored and integrated into clinical tools that can be used to identify those at highest risk [1,6–9]. However, whether these same predictors identify those at risk of readmission among the subset discharged on OAC therapy has not been extensively investigated. Prior studies have shown that patients undergoing PCI on chronic OAC therapy have a higher burden of cardiovascular disease, experience greater post-procedure bleeding complications, and have increased risk of long-term mortality compared to patients not on chronic OAC therapy [10–12]. Following discharge after PCI, patients on chronic OAC therapy were also found to experience a significantly higher 90-day readmission rate [11] The details of why these patients were rehospitalized, however, remain unclear.

Given the association between OAC therapy and risk of readmission, our study was designed to accomplish two main goals: 1) understand the reasons for 30-day readmission among patients who are discharged on OACs after index PCI; and 2) determine predictors of 30-day readmission in this patient group and assess whether these vary from those not on OAC. This information may in turn be used to assist clinicians in developing interventions targeted towards reducing post-PCI readmissions among patients on OAC therapy.

## Materials and methods

### Study population

Partners HealthCare is an integrated health care system founded by the Massachusetts General Hospital and Brigham and Women’s Hospital, consisting of multiple hospitals, community health centers, and ambulatory practices. For this analysis, we included consecutive patients undergoing PCI at two Partners HealthCare medical centers (Massachusetts General Hospital or Brigham and Women’s Hospital) between June 2009 and September 2014. If more than one PCI was performed within 30 days, only the first was included in the analysis. No other exclusion criteria were utilized. Due to the retrospective nature of this analysis, the Partners HealthCare Institutional Review Board waived the requirement for informed consent, and data were fully anonymized for statistical analysis.

### Covariates

Clinical and procedural characteristics for all PCIs were obtained from institutional registry data, which was derived from the National Cardiovascular Data Registry’s CathPCI Registry data collection form. The data elements in the CathPCI Registry form can be found online (https://www.ncdr.com/WebNCDR/docs/default-source/public-data-collection-documents/cathpci_v4_datacollectionform_4-4.pdf?sfvrsn=2) [13].

### Exposure of interest

Determination of oral anticoagulation status at the time of discharge from index PCI was based on the discharge medication list or discharge summary. Type of OAC therapy, concomitant use of antiplatelet therapies, and doses used were collected as well.

### Outcomes

The primary outcome in the analysis was all-cause, 30-day readmission. All readmissions to any of the hospitals affiliated with Partners Healthcare within 30 days of the index PCI were identified. For those with >1 readmission, only the first was used for the analysis. All patients in the study had at least 30 days of follow-up after discharge following PCI. For those readmitted, the following data were collected manually by two physicians: reason for readmission, type of oral anticoagulant and dose at readmission and discharge, and types of antiplatelet agents and doses at readmission and discharge. If bleeding was the reason for readmission, location and severity of the bleeding based on the Bleeding Academic Research Consortium (BARC) classification [14] were documented. If chest pain was the reason for readmission, the cause of chest pain was documented as ST-elevation myocardial infarction (STEMI), non-ST-segment elevation myocardial infarction (NSTEMI), unstable angina, stable angina, pericarditis, or noncardiac chest pain. Noncardiac chest pain was defined as chest pain resulting from noncardiac-related etiologies, such as gastrointestinal or musculoskeletal. Among those readmitted, we also evaluated in-hospital mortality and length of stay in days during the readmission.

### Statistical analysis

Continuous data are shown as mean ± standard deviation and analyzed using Wilcoxon rank sum tests or Student’s t tests, and categorical variables are shown as n (%) and analyzed with chi-square tests. Multivariable logistic regression models were created to determine independent predictors of 30-day readmission and bleeding among all readmitted patients. In addition, a similar model to determine independent predictors of 30-day readmissions was developed among the cohort of patients discharged on OACs after index PCI. This model was created to identify predictors of readmission specific to those discharged on OACs. We followed the recommendation of limiting the number of potential predictor variables to 1 per 10 events (10 events per variable) [15]. All candidate variables were retained in the final model with no selection procedures used.

Candidate variables included in the logistic regression readmission models were selected based on clinical knowledge and prior studies [1,6,7,16,17]. For the total study population, these included the following: OAC at discharge, age, gender, ethnicity, insurance type, admission status, comorbidities (heart failure, diabetes mellitus, chronic lung disease, chronic kidney disease [stratified as glomerular filtration rate (GFR) 30–60 mL/minute or GFR<30 mL/min/dialysis], peripheral arterial disease (defined as disease involving the upper and lower extremity, mesenteric, renal, and abdominal aortic system vasculature), prior PCI, prior coronary artery bypass graft surgery), procedural characteristics (cardiogenic shock on presentation, nonelective PCI [defined as urgent/emergent/salvage], use of drug-eluting stent), postprocedural complications (postprocedural bleed, index hospitalization length of stay greater than 5 days), and discharge characteristics (discharge to home, beta-blocker prescribed on discharge). For the readmitted patients on OAC therapy, a more limited number of variables were chosen *a priori* in order to avoid model overfitting. The variables included those variables used in the full readmission model, with the exception of OAC on discharge, gender, admission status, cardiogenic shock on presentation, and discharge characteristics (discharge to home, beta-blocker prescribed on discharge). For the model created to determine predictors of bleeding-related readmissions in the overall readmitted population, in order to avoid model overfitting, the CathPCI bleeding risk score, previously developed to determine patient’s risk of bleeding after PCI, was used as a candidate variable [18]. Other variables included in the bleeding predictors model are listed in S1 Fig, as are the variables included in the CathPCI bleeding risk score. All analysis was done with SAS version 9.4 (Cary, NC), with a p-value of 0.05 marking statistical significance.

## Results

### Clinical characteristics of patients discharged on OAC therapy after index PCI

From 2009 through 2014, 9,357 patients underwent PCI and survived to discharge. Of these patients, 1,110 (11.9%) were discharged on OACs. Characteristics of the study cohort stratified by discharge OAC status are shown in Table 1. Patients discharged on OACs were older and generally had a higher burden of cardiovascular and noncardiovascular diseases. During the index hospitalization, patients ultimately discharged on OACs presented more often with STEMI, experienced higher rates of cardiogenic shock within 24 hours of PCI, and underwent higher rates of emergent PCI. Drug-eluting stents were less commonly used in patients discharged on OACs. Following PCI, patients ultimately discharged on OACs had higher rates of stroke (0.81% vs 0.34%; p = 0.02), longer lengths of stay (5.2 ± 7.0 days vs 2.4 ± 3.6 days, p<0.01), and higher rates of bleeding events (12.3% vs 5.1%, p<0.01) during the hospitalization. Of bleeding events in the OAC group, the majority were due to non-access site bleeding (84.6%). Clinical characteristics of all patients discharged on OACs, including reasons for anticoagulation, are included in the Supporting Information (S1 Table).

**Table 1:** Baseline and procedural characteristics of all patients discharged after percutaneous coronary intervention (PCI), stratified by discharge OAC status.

Characteristic	OAC at Discharge (n = 1110)	No OAC at Discharge (n = 8247)	*P* Value
Age (years, mean ± SD)	69.5 ± 12.0	65.4 ± 12.0	<0.01
Male	821 (74.0)	5978 (72.5)	0.30
BMI (kg/m^2^, mean ± SD)	29.4 ± 6.5	29.2 ± 5.9	0.15
White	1016 (91.5)	7496 (90.9)	0.49
Hypertension	934 (84.1)	6759 (82.0)	0.07
Dyslipidemia	1021 (92.0)	7717 (93.6)	0.05
Diabetes mellitus	417 (37.6)	2834 (34.4)	0.04
Renal failure (currently on dialysis or creatinine > 2 mg/dL)	85 (7.7)	408 (5.0)	<0.01
Current or recent smoker (within 1 year)	148 (13.3)	1497 (18.2)	<0.01
Family history of premature CAD	183 (16.5)	2002 (24.3)	<0.01
Prior MI	483 (43.5)	2858 (34.7)	<0.01
Prior PCI	399 (36.0)	3213 (39.0)	0.05
Prior CABG	298 (26.9)	1545 (18.7)	<0.01
Prior valve surgery or procedure	116 (10.5)	150 (1.8)	<0.01
Cerebrovascular disease	262 (23.6)	1105 (13.4)	<0.01
Prior heart failure	364 (32.8)	1104 (13.4)	<0.01
Peripheral arterial disease	227 (20.5)	1273 (15.4)	<0.01
Chronic lung disease	197 (17.8)	1081 (13.1)	<0.01
Insurance			
Medicare	655 (59.0)	3486 (42.3)	<0.01
Medicaid	42 (3.8)	496 (6.0)	<0.01
Private	422 (38.0)	4181 (50.7)	<0.01
None	21 (1.9)	203 (2.5)	0.24
Presentation type			
Stable angina	160 (14.4)	1775 (21.5)	<0.01
Unstable angina	265 (23.9)	2374 (28.8)	<0.01
NSTEMI	251 (22.6)	1929 (23.4)	0.56
STEMI	192 (17.3)	1003 (12.2)	<0.01
No symptoms / no angina	211 (19.0)	1021 (12.4)	<0.01
Symptoms unlikely to be ischemic	31 (2.8)	145 (1.8)	0.02
PCI status			
Elective	317 (28.6)	2908 (35.3)	<0.01
Urgent	553 (49.8)	4071 (49.4)	0.78
Emergency	239 (21.5)	1251 (15.2)	<0.01
Salvage	1 (0.1)	17 (0.2)	0.41
Cardiogenic shock within 24 hours	44 (4.0)	95 (1.2)	<0.01
DES placed	457 (41.2)	5730 (69.5)	<0.01
Post-procedure complications			
CVA	9 (0.8)	28 (0.3)	0.02
MI	30 (2.7)	231 (2.8)	0.85
Bleeding event	136 (12.3)	423 (5.1)	<0.01
Non-access site bleeding	115 (84.6)	335 (79.2)	<0.01
Access site bleeding	21 (15.4)	88 (20.8)	0.02
Length of stay (days, mean ± SD)	5.2 ± 7.0	2.4 ± 3.6	<0.01
Readmitted after 30 days	143 (12.9)	549 (6.7)	<0.01
Indication for oral anticoagulation among readmitted patients			
Nonvalvular atrial fibrillation	591 (53.2)	—	—
Left ventricle thrombus	124 (11.2)	—	—
Valvular atrial fibrillation	93 (8.4)	—	—
Pulmonary embolus	82 (7.4)	—	—
Deep vein thrombosis	87 (7.4)	—	—
Left ventricle aneurysm	60 (5.4)	—	—
Cardioembolic stroke	44 (4.0)	—	—
Atrial flutter	77 (6.9)	—	—
Hypercoagulable syndrome	63 (5.7)	—	—
Valvular disease	42 (3.8)	—	—
Other	49 (4.4)	—	—
OAC at discharge from index PCI			
Warfarin	1047 (94.3)	—	—
Dabigatran	29 (2.6)	—	—
Rivaroxaban	23 (2.1)	—	—
Apixaban	4 (0.4)	—	—
P2Y_12_ inhibitor at discharge from index PCI			
Clopidogrel	1007 (90.7)	7255 (88.0)	<0.01
Ticagrelor	28 (2.5)	448 (5.4)	<0.01
Prasugrel	14 (1.3)	315 (3.8)	<0.01

Data are shown as n (%) except where otherwise noted. BMI, body mass index; CABG, coronary artery bypass graft surgery; CAD, coronary artery disease; CVA, cerebrovascular accident; DES, drug eluting stent; MI, myocardial infarction; NSTEMI, non-ST-segment elevation myocardial infarction; OAC, oral anticoagulant; PCI, percutaneous coronary intervention; SD, standard deviation; STEMI, ST-segment elevation myocardial infarction.

### Clinical characteristics of readmitted patients and reasons for readmission

During the study period, 692 patients were readmitted within 30 days. Of the readmitted patients, 20.7% (n = 143) were on OACs, with a readmission rate of 12.9% among all patients discharged on OACs. In comparison, 79.3% (n = 549) of the readmitted patients had not been discharged on OACs, with a readmission rate of 6.7% among all patients not discharged on OACs. Characteristics of those readmitted are shown in Table 2. Chronic anticoagulant therapy, defined as OAC use within 30 days preceding index PCI, was used by 67.7% of all patients on OACs and by 50.4% of those readmitted. Readmitted patients on OACs were older, had higher rates of prior valve surgery, longer lengths of stay after index PCI, and higher rates of overall bleeding during the index PCI hospitalization compared to readmitted patients not discharged on OACs.

**Table 2:** Baseline and procedural characteristics of patients readmitted within 30 days after percutaneous coronary intervention (PCI).

Characteristic	OAC at Discharge (n = 143)	No OAC at Discharge (n = 549)	*P* Value
Age (years, mean ± SD)	70.1 ± 12.2	67.5 ± 12.7	0.03
Male	100 (69.9)	357 (65.0)	0.27
BMI (kg/m^2^, mean ± SD)	29.3 ± 7.0	28.9 ± 6.5	0.53
White	132 (92.3)	482 (87.8)	0.13
Hypertension	124 (86.7)	469 (85.4)	0.70
Dyslipidemia	128 (89.5)	516 (94.0)	0.06
Diabetes mellitus	44 (30.8)	227 (41.4)	0.02
Renal failure (currently on dialysis or creatinine > 2 mg/dL)	18 (12.6)	59 (10.8)	0.53
Current or recent smoker (within 1 year)	20 (14.0)	99 (18.0)	0.25
Family history of premature CAD	21 (14.7)	117 (21.3)	0.08
Prior MI	56 (39.2)	209 (38.1)	0.81
Prior PCI	35 (24.5)	182 (33.2)	0.05
Prior CABG	30 (21.0)	119 (21.7)	0.86
Prior valve surgery or procedure	20 (14.0)	14 (2.55)	<0.01
Cerebrovascular disease	34 (23.8)	94 (17.1)	0.07
Prior heart failure	44 (30.8)	131 (23.9)	0.09
Peripheral arterial disease	41 (28.7)	123 (22.4)	0.12
Chronic lung disease	29 (20.3)	99 (18.0)	0.54
Insurance			
Medicare	85 (59.4)	297 (54.1)	0.25
Medicaid	8 (5.6)	39 (7.1)	0.52
Private	53 (37.1)	211 (38.4)	0.76
None	1 (0.7)	13 (2.4)	0.21
Presentation type			
Stable angina	11 (7.7)	60 (10.9)	0.26
Unstable angina	33 (23.1)	161 (29.3)	0.14
NSTEMI	37 (25.9)	163 (29.7)	0.37
STEMI	37 (25.9)	83 (15.1)	<0.01
No symptoms / no angina	19 (13.3)	73 (13.3)	1.00
Symptoms unlikely to be ischemic	6 (4.2)	9 (1.6)	0.06
PCI status			
Elective	23 (16.1)	110 (20.0)	0.29
Urgent	77 (53.9)	335 (61.0)	0.12
Emergency	42 (29.4)	102 (18.6)	<0.01
Salvage	1 (0.7)	2 (0.4)	0.59
Cardiogenic shock within 24 hours	9 (6.3)	7 (1.3)	<0.01
DES placed	54 (37.8)	320 (58.3)	<0.01
Post-procedure complications			
CVA	1 (0.7)	6 (1.1)	0.68
MI	3 (2.1)	21 (3.8)	0.31
Bleeding event	23 (16.1)	50 (9.1)	0.02
Non-access site bleeding	18 (78.3)	41 (82.0)	0.05
Access site bleeding	5 (21.7)	9 (18.0)	0.16
Length of stay (days, mean ± SD)	7.5 ± 11.1	4.1 ± 6.0	<0.01
Indication for oral anticoagulation among readmitted patients			
Nonvalvular atrial fibrillation	68 (47.6)	—	—
Left ventricle thrombus	19 (13.3)	—	—
Valvular atrial fibrillation	13 (9.1)	—	—
Pulmonary embolus	12 (8.4)	—	—
Deep vein thrombosis	11 (7.7)	—	—
Left ventricle aneurysm	11 (7.7)	—	—
Cardioembolic stroke	9 (6.3)	—	—
Atrial flutter	7 (4.9)	—	—
Hypercoagulable syndrome	7 (4.9)	—	—
Valvular disease	7 (4.9)	—	—
Other	5 (3.5)	—	—
OAC at discharge from index PCI			
Warfarin	138 (96.5)	—	—
Rivaroxaban	3 (2.1)	—	—
Apixaban	1 (0.7)	—	
Dabigatran	0 (0.0)	—	—
P2Y_12_ inhibitor at discharge from index PCI			
Clopidogrel	125 (87.4)	479 (87.3)	0.96
Ticagrelor	4 (2.8)	30 (5.5)	0.19
Prasugrel	1 (0.7)	9 (1.6)	0.40

Data are shown as n (%) except where otherwise noted. BMI, body mass index; CABG, coronary artery bypass graft surgery; CAD, coronary artery disease; CVA, cerebrovascular accident; DES, drug eluting stent; MI, myocardial infarction; NSTEMI, non-ST-segment elevation myocardial infarction; OAC, oral anticoagulant; PCI, percutaneous coronary intervention; SD, standard deviation; STEMI, ST-segment elevation myocardial infarction.

The most common reasons for readmission, stratified by discharge OAC status, are shown in Table 3. The complete list of reasons for readmission is shown in S2 Table. The most common cause of readmission for both groups was chest pain syndromes (34.4% not discharged on OACs; 21.7% discharged on OACs), the majority of which were for stable and unstable angina (44.4% not discharged on OACs; 41.9% discharged on OACs). Following this, post-discharge bleeding was the next most common reason for readmission among patients discharged on OACs (14.0%), which occurred with greater frequency compared to those not discharged on OACs (6.0%, p<0.01). Among OAC patients, gastrointestinal hemorrhage was the most frequent cause of bleeding (85.0%). The majority of bleeds were classified as BARC 3a (55.0%), followed by type 2 (25.0%) and 3b (20.0%) in the OAC population. No fatal bleeding events occurred among all patients. Details regarding the BARC classification for all bleeding-related readmissions are shown in the Supporting Information (S3 Table). During rehospitalization, patients discharged on OACs relative to those not on OACs experienced a higher unadjusted rate of mortality (6.3% vs 1.8%, respectively, p<0.01) and longer lengths of stay (6.4 ± 7.1 days vs 4.9 ± 6.8 days, p = 0.02). Among readmitted patients on OACs, there were no significant differences in outcomes observed after stratifying patients by chronic anticoagulant status (S4 Table).

**Table 3:** Reasons for 30-day readmissions.

Reasons	OAC at Discharge (n = 143)	No OAC at Discharge (n = 549)	*P* Value
Chest pain syndromes	31 (21.7)	189 (34.4)	<0.01
Stable angina	3 (9.7)	21 (11.1)	0.31
Unstable angina	10 (32.3)	63 (33.3)	0.12
NSTEMI	2 (6.5)	32 (16.9)	0.03
STEMI	1 (3.2)	0 (0.0)	0.05
Noncardiac chest pain	10 (32.3)	67 (35.4)	0.08
Pericarditis	4 (12.9)	5 (2.7)	0.08
Bleeding	20 (14.0)	33 (6.0)	<0.01
Gastrointestinal	17 (85.0)	23 (69.7)	<0.01
Access site	1 (5.0)	2 (6.1)	0.59
Genitourinary	0 (0.0)	3 (9.1)	0.38
Intracranial	0 (0.0)	1 (3.0)	0.61
Other	2 (10.0)	4 (12.1)	0.44
Epistaxis	1 (5.0)	0 (0.0)	—
Skin/MSK	1 (5.0)	2 (6.1)	—
Pulmonary	0 (0.0)	2 (6.1)	—
Congestive heart failure	19 (13.3)	53 (9.7)	0.21
Elective peripheral procedure or surgery	6 (4.2)	16 (2.9)	0.44
Stroke or TIA (not related to PCI)	6 (4.2)	9 (1.6)	0.06
Atrial fibrillation	5 (3.5)	9 (1.6)	0.16
Syncope or presyncope	5 (3.5)	26 (4.7)	0.52
Aortic stenosis	4 (2.8)	7 (1.3)	0.19
Stent thrombosis	4 (2.8)	14 (2.6)	0.87
Pneumonia	3 (2.1)	8 (1.5)	0.59
Vascular complication of PCI (aneurysm, fistula)	3 (2.1)	4 (0.7)	0.14
Venous thromboembolism	3 (2.1)	3 (0.6)	0.07
Ventricular tachycardia	3 (2.1)	6 (1.1)	0.34
Bradycardia	2 (1.4)	1 (0.2)	0.05
Elective CABG	2 (1.4)	11 (2.0)	0.64
Hypotension	2 (1.4)	5 (0.9)	0.60
Bacteremia or endocarditis	1 (0.7)	4 (0.7)	0.97
Cholecystitis, gastroenteritis, colitis/enteritis, pancreatitis, cholangitis, or abdominal pain	1 (0.7)	23 (4.2)	0.04
Elective ICD/CRT placement	1 (0.7)	3 (0.6)	0.83
Sepsis	1 (0.7)	7 (1.3)	0.57
Staged PCI without new symptoms	1 (0.7)	10 (1.8)	0.34
Viral infection, URI, bronchitis	1 (0.7)	3 (0.6)	0.83
Anxiety, depression, or panic attack	0 (0.0)	2 (0.4)	0.47
Chronic obstructive pulmonary disease	0 (0.0)	3 (0.6)	0.38
Fever	0 (0.0)	4 (0.7)	0.31
Renal failure	0 (0.0)	9 (1.6)	0.12
Rhabdomyolysis	0 (0.0)	2 (0.4)	0.47
Urinary tract infection or urosepsis	0 (0.0)	8 (1.5)	0.15
Other	16 (11.2)	73 (13.3)	0.50

Data are shown as n (%) except where otherwise noted. CABG, coronary artery bypass graft surgery; CRT, cardiac resynchronization therapy; ICD, implantable cardioverter-defibrillator; MSK, musculoskeletal; NSTEMI, non-ST-segment elevation myocardial infarction; OAC, oral anticoagulant; PCI, percutaneous coronary intervention; STEMI, ST-segment elevation myocardial infarction; TIA, transient ischemic attack; URI, upper respiratory tract infection.

### Changes to medication regimens at the time of discharge from the readmission

Of the 143 patients on OACs at the time of discharge from the index PCI and who were readmitted, 130 (90.9%) were on triple therapy (defined as an OAC, aspirin, and a P2Y_12_ inhibitor). Most patients were discharged from the index hospitalization on warfarin (96.5%), followed by rivaroxaban (2.1%) and apixaban (0.7%). At the time of discharge from the readmission, 101 (75.4%) remained on OACs, with 98 (97.0%) on warfarin and 84 (83.2%) receiving triple therapy. Of the patients whose OAC therapy was discontinued at the time of discharge from the readmission, 78.8% were discharged on dual antiplatelet therapy, 12.1% on aspirin alone, and 3.0% on a P2Y_12_ inhibitor alone. Complete details regarding medication regimens are shown in the Supporting Information (S5 Table).

### Predictors of readmission and bleeding

Among readmitted patients, OAC use was found in adjusted analysis to be associated with a 1.46-fold increased odds of readmission (95% confidence interval [CI] 1.18–1.80, p<0.01). Other significant independent risk factors for readmission included Medicare insurance (odds ratio [OR] 1.25, 95% CI 1.05–1.50, p = 0.01), prior heart failure (OR 1.33, 95% CI 1.08–1.64, p<0.01), chronic kidney disease (OR 1.9, 95% CI 1.31–2.75, p<0.01), nonelective PCI (OR 1.81, 95% CI 1.32–2.50, p<0.01), and index hospitalization length of stay greater than 5 days (OR 1.52, 95% CI 1.21–1.92, p<0.01). Placement of a drug-eluting stent (DES) during the index PCI was associated with a lower risk of readmission (OR 0.79, 95% CI 0.67–0.93, p<0.01), as was history of a prior PCI (OR 0.73, 95% CI 0.61–0.87, p<0.01). Results of the full model can be found in S2 Fig.

**Fig 1:**
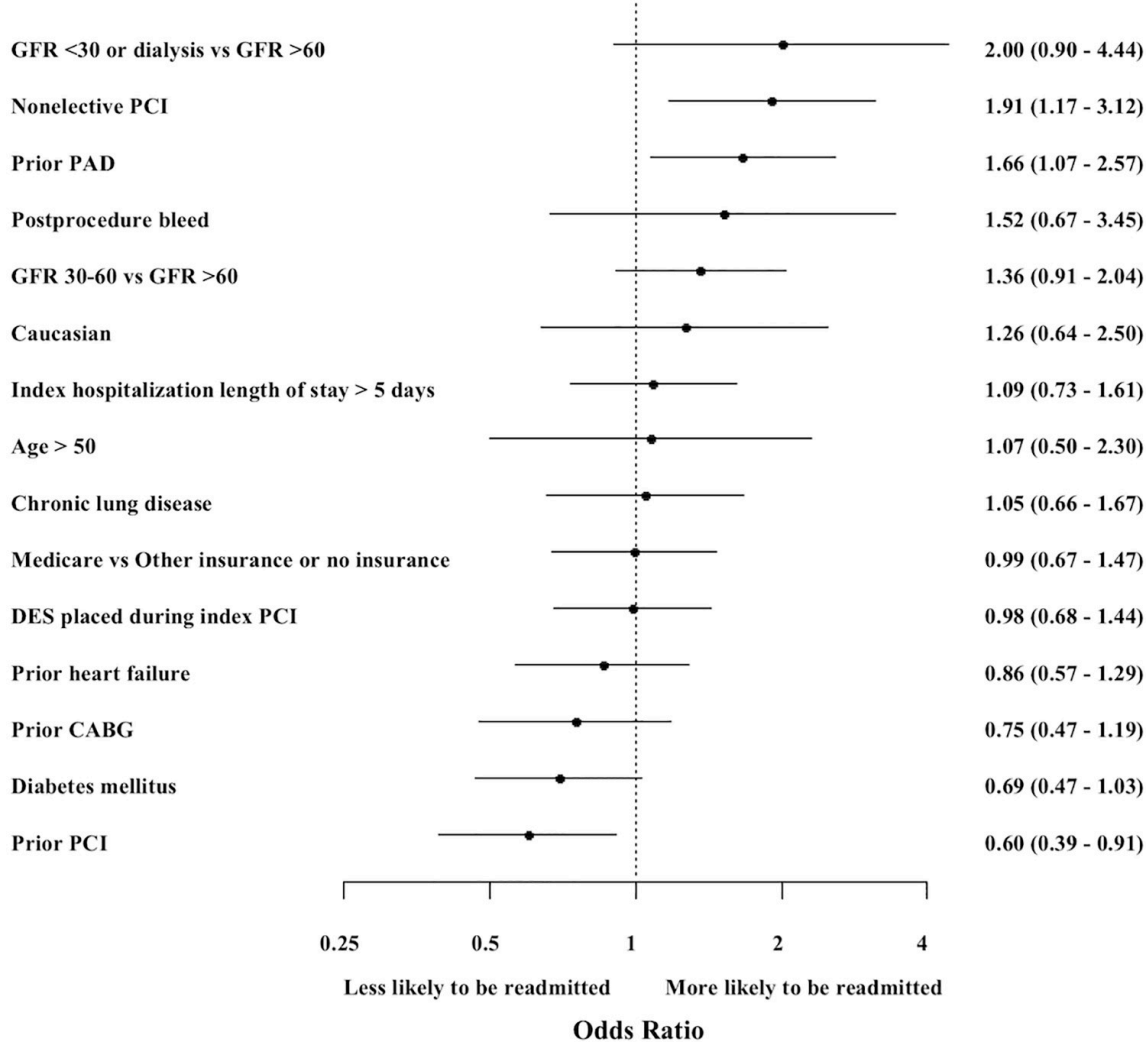
Predictors of readmission among patients discharged on oral anticoagulant (OAC) therapy. CABG, coronary artery bypass graft surgery; DES, drug eluting stent; GFR, glomerular filtration rate (mL/minute); LOS, length of stay; OAC, oral anticoagulant; PAD, peripheral arterial disease; PCI, percutaneous coronary intervention.

Among the subset of patients discharged on OACs following the index PCI, predictors of readmission included nonelective PCI (OR 1.91, 95% 1.17–3.12, p<0.01) and history of peripheral arterial disease (PAD) (OR 1.66, 95% CI 1.07–2.57, p = 0.02), whereas history of prior PCI was associated with a reduced risk of readmission (OR 0.60, 95% CI 0.39–0.91, p = 0.02) (Fig 1). In addition, in the group of patients who were readmitted for bleeding-related reasons, OAC use at the time of discharge from index PCI was associated with increased risk of bleeding (OR 3.37, 95% CI 1.85–6.14, p<0.01), as was an increasing CathPCI bleeding risk score (OR 1.03 per every 1% increase in bleeding risk, 95% CI 1.01–1.05, p<0.01) and a history of heart failure (OR 1.90, 95% CI 1.01–3.57, p = 0.05). A full list of variables is provided in S1 Fig.

## Discussion

In this study of over 1,100 patients on OAC therapy after PCI, we assessed the reasons and predictors for early readmissions. We found that patients discharged on OACs following PCI are more commonly readmitted within 30 days than patients not discharged on OACs, with chest pain (21.7%) and bleeding (14.0%) representing the major causes. Bleeding-related readmissions were significantly greater among OAC patients, of which the majority were due to gastrointestinal bleeding. Readmissions were associated with prolonged lengths of stay and a high unadjusted mortality rate in the OAC population. OAC use after discharge from index PCI was found to be a strong independent predictor of readmission among all readmitted patients, whereas among patients on OACs, peripheral arterial disease and nonelective index PCI were independently associated with 30-day readmission.

Patients who were discharged on OACs after PCI were readmitted for a diverse group of reasons, yet similar to prior studies among all-comers, chest pain was the primary reason for readmission [1,9]. However, we found that bleeding was the next most frequent cause of readmission, occurring at more than twice the rate compared with those not on OACs. While prior studies have not fully described reasons for readmissions among stented patients on OACs, analyses among all-comers after PCI have consistently found bleeding to be a less frequent reason for readmission. For instance, in an analysis by Wasfy et al., nonaccess site bleeding accounted for 3.7% of all 30-day readmissions, the fifth most common reason [9]. Similarly, McNeely et al. reported a 30-day readmission rate of gastrointestinal bleeding of only 1.8% [8].

While bleeding-related readmissions are not unexpected among patients on OACs, it is important to recognize this as a major cause of early readmission after PCI given both the morbidity and costs related to these events. Bleeding-related readmissions may influence patient outcomes. Ko et al. concluded that bleeding-related readmissions after PCI are associated with increased risks of death and myocardial infarction among all patients, and this may be true for patients on OACs as well [19]. In addition, a prior analysis from the Dual Antiplatelet Therapy trial found that late bleeding after PCI has a poor prognosis, with an 18.1-fold increased hazard of mortality, nearing that of late ischemic events [20]. Studies have also shown that bleeding events related to anticoagulation are associated with high healthcare expenditures, and a 2011 study found that bleeding events were found to raise costs of patients with nonvalvular atrial fibrillation on OAC therapy by $30–45,000 per patient per year [21,22]. Although we did not collect data regarding diagnostic or therapeutic procedures that patients underwent during bleeding-related readmissions, it is reasonable to hypothesize that these patients may undergo diagnostic and therapeutic procedures such as upper endoscopies or colonoscopies during the evaluation of gastrointestinal bleeding, which can lead to escalating costs.

Among all patients discharged after PCI, OAC use was found to be independently associated with 30-day readmission and bleeding events, which has been suggested by other studies [21]. This may provide a definable target for intervention. After PCI, it is critical that the physician reevaluate the need for oral anticoagulation, and carefully assess a patient’s ischemic and bleeding risk, to make an individualized decision regarding the optimal combination of antithrombotic and antiplatelet therapy. In our analysis, we specifically showed that the CathPCI bleeding risk score [18] is an independent predictor of bleeding-related readmission, and clinicians can also use one of many available risk scores to help assess a patient’s ischemic and bleeding risks [23–26]. In addition, several trials have suggested that following PCI, an OAC and a single antiplatelet agent may be more appropriate than triple therapy [27–29], and prioritizing novel oral anticoagulants, which have been shown to provide a lower risk of bleeding compared to warfarin, may be an additional strategy [28–29]. Lastly, our study identified PAD and nonelective indications for PCI as additional risk factors for readmission among stented patients on OACs, which provides readily available patient characteristics to identify those at highest risk. Readmissions may be also reduced if the transition of care for patients on OACs is optimized prior to discharge, and programs emphasizing extensive patient education and close post-discharge follow-up appointments have previously shown reduction in readmissions after PCI [2,30].

Our study has limitations. First, warfarin was the most common oral anticoagulant prescribed among our patient population, and our results may not be generalizable to those on novel oral anticoagulants. Second, data on readmissions were obtained from within our healthcare system. Although a previous study has demonstrated that the majority of post-PCI patients are readmitted to the procedural hospital [9], our analysis is unable to account for readmissions that occurred at hospitals outside of our healthcare system. Third, the overall study population included predominantly white, male patients, and as such, our generalizability is limited among patients undergoing PCI with other characteristics. Fourth, the number of readmissions and bleeding events in the OAC group was small, which limits the number of variables we were able to include in our regression models. Finally, data regarding the use of agents such as proton pump inhibitors and H2-receptor antagonists were not available.

## Conclusions

In conclusion, we found that patients on OACs are commonly readmitted within 30 days following PCI, with bleeding representing a major cause. These readmissions were associated with a high risk of mortality. Peripheral arterial disease and nonelective index PCI were important predictors of readmission in the OAC group. Therefore, patients on OACs merit close monitoring following discharge after PCI, and further research is required to determine how to prevent readmissions and bleeding-related events among this complex cohort of patients.

## Supporting information

S1 Fig a) Predictors of bleeding-related readmissions among all patients. b) Components of the CathPCI bleeding risk score. BMI, body mass index; Hb, hemoglobin; OAC, oral anticoagulant; PCI, percutaneous coronary intervention; STEMI, ST-segment elevation myocardial infarction.
(TIFF)

S2 Fig Predictors of readmission among all patients. CABG, coronary artery bypass graft surgery; DES, drug eluting stent; GFR, glomerular filtration rate (mL/minute); OAC, oral anticoagulant; PAD, peripheral arterial disease; PCI, percutaneous coronary intervention.
(TIFF)

S1 Table Characteristics of patients on oral anticoagulant (OAC) therapy, stratified by readmission status. Data are shown as n (%) except where otherwise noted. BMI, body mass index; CABG, coronary artery bypass graft surgery; CAD, coronary artery disease; CVA, cerebrovascular accident; DES, drug eluting stent; MI, myocardial infarction; NSTEMI, non-ST-segment elevation myocardial infarction; OAC, oral anticoagulant; PCI, percutaneous coronary intervention; SD, standard deviation; STEMI, ST-segment elevation myocardial infarction.
(DOCX)

S2 Table Complete list of reasons for readmission. Data are shown as n (%) except where otherwise noted. CABG, coronary artery bypass graft surgery; CRT, cardiac resynchronization therapy; ICD, implantable cardioverter-defibrillator; MSK, musculoskeletal; NSTEMI, non-ST-segment elevation myocardial infarction; OAC, oral anticoagulant; PCI, percutaneous coronary intervention; STEMI, ST-segment elevation myocardial infarction; TIA, transient ischemic attack; URI, upper respiratory tract infection.
(DOCX)

S3 Table BARC classification of all bleeding events in readmitted patients. Data are shown as n (%). BARC, Bleeding Academic Research Consortium; OAC, oral anticoagulant.
(DOCX)

S4 Table Outcomes of patients on OACs during readmission, stratified by chronicity of OAC use. Data are shown as n (%) except where otherwise noted. CABG, coronary artery bypass graft surgery; CRT, cardiac resynchronization therapy; ICD, implantable cardioverter-defibrillator; MSK, musculoskeletal; NSTEMI, non-ST-segment elevation myocardial infarction; OAC, oral anticoagulant; PCI, percutaneous coronary intervention; STEMI, ST-segment elevation myocardial infarction; TIA, transient ischemic attack; URI, upper respiratory tract infection.
(DOCX)

S5 Table Medication regimens for patients discharged on oral anticoagulant therapy and readmitted. Data are shown as n (%). ASA, aspirin; OAC, oral anticoagulant.
(DOCX)

## References

[1] Kwok CS, Hulme W, Olier I, Holroyd E, Mamas MA. Review of early hospitalisation after percutaneous coronary intervention. Int J Cardiol. 2017; 227:370–377. 10.1016/j.ijcard.2016.11.050 27839805

[2] Tanguturi VK, Temin E, Yeh RW, Thompson RW, Rao SK, Mallick A, et al Clinical Interventions to Reduce Preventable Hospital Readmission After Percutaneous Coronary Intervention. Circ Cardiovasc Qual Outcomes. 2016; 9:600–604. 10.1161/CIRCOUTCOMES.116.003086 27553598

[3] Readmissions Reduction Program (HRRP). Centers for Medicare & Medicaid Services. Available at https://www.cms.gov/Medicare/Medicare-Fee-for-Service-Payment/AcuteInpatientPPS/Readmissions-Reduction-Program.html. Accessed December 28, 2017.

[4] Wasfy JH, Zigler CM, Choirat C, Wang Y, Dominici F, Yeh RW. Readmission Rates After Passage of the Hospital Readmissions Reduction Program: a Pre–Post analysis. Ann Intern Med. 2017;166:324–331. 10.7326/M16-0185 28024302 PMC5507076

[5] BPCI Advanced. Centers for Medicare & Medicaid Services. Available at https://innovation.cms.gov/initiatives/bpci-advanced. Accessed April 11, 2018.

[6] Wasfy JH, Rosenfield K, Zelevinsky K, Sakhuja R, Lovett A, Spertus JA, et al A Prediction Model to Identify Patients at High Risk for 30-Day Readmission After Percutaneous Coronary Intervention. Circ Cardiovasc Qual Outcomes. 2013; 6:429–435. 10.1161/CIRCOUTCOMES.111.000093 23819957

[7] Ricciardi MJ, Selzer F, Marroquin OC, Holper EM, Venkitachalam L, Williams DO, et al Incidence and Predictors of 30-Day Hospital Readmission Rate Following Percutaneous Coronary Intervention (from the National Heart, Lung, and Blood Institute Dynamic Registry). Am J Cardiol. 2012; 110:1389–1396. 10.1016/j.amjcard.2012.07.002 22853982 PMC3483468

[8] McNeely C, Markwell S, Vassileva CM. Readmission after inpatient percutaneous coronary intervention in the Medicare population from 2000 to 2012. Am Heart J. 2016; 179:195–203. 10.1016/j.ahj.2016.07.002 27595697

[9] Wasfy JH, Strom JB, O’Brien C, Zai AH, Luttrell J, Kennedy KF, et al Causes of Short-Term Readmission After Percutaneous Coronary Intervention. Circ Cardiovasc Interv. 2014; 7:97–103. 10.1161/CIRCINTERVENTIONS.113.000988 24425587

[10] Oudot A, Steg PG, Danchin N, Dentan G, Zeller M, Sicard P, et al Impact of chronic oral anticoagulation on management and outcomes of patients with acute myocardial infarction: data from the RICO survey. Heart. 2006; 92:1077–1083. 10.1136/hrt.2005.074070 16387830 PMC1861111

[11] Secemsky EA, Butala NM, Kartoun U, Mahmood S, Wasfy JH, Kennedy KF, et al Use of Chronic Oral Anticoagulation and Associated Outcomes Among Patients Undergoing Percutaneous Coronary Intervention. J Am Heart Assoc. 2016; 5:e004310 10.1161/JAHA.116.004310 27792650 PMC5121523

[12] Rubboli A, Colletta M, Valencia J, Capecchi A, Franco N, Zanolla L, et al Periprocedural management and in-hospital outcome of patients with indication for oral anticoagulation undergoing coronary artery stenting. J Interv Cardiol. 2009; 22:390–397. 10.1111/j.1540-8183.2009.00468.x 19453820

[13] Data Collection. National Cardiovascular Data Registry. Available at: https://www.ncdr.com/WebNCDR/docs/default-source/public-data-collection-documents/cathpci_v4_datacollectionform_4-4.pdf?sfvrsn=2CollectionForm.ashx. Accessed February 7, 2018.

[14] Mehran R, Rao SV, Bhatt DL, Gibson CM, Caixeta A, Eikelboom J, et al Standardized bleeding definitions for cardiovascular clinical trials: a consensus report from the Bleeding Academic Research Consortium. Circulation. 2011; 123:2736–2747. 10.1161/CIRCULATIONAHA.110.009449 21670242

[15] Peduzzi P, Concato J, Kemper E, Holford TR, Feinstein AR. A simulation study of the number of events per variable in logistic regression analysis. J Clin Epidemiol. 1996; 49:1373–1379. 8970487 10.1016/s0895-4356(96)00236-3

[16] Khawaja FJ, Shah ND, Lennon RJ, Slusser JP, Alkatib AA, Rihal CS, et al Factors Associated With 30-Day Readmission Rates After Percutaneous Coronary Intervention. Arch Intern Med. 2012; 172:112–117. 10.1001/archinternmed.2011.569 22123752 PMC3688066

[17] Minges KE, Herrin J, Fiorilli PN, Curtis JP. Development and Validation of A Simple Risk Score to Predict 30-Day Readmission after Percutaneous Coronary Intervention in a Cohort of Medicare Patients. Catheter Cardiovasc Interv. 2017; 89:955–963. 10.1002/ccd.26701 27515069 PMC5397364

[18] Rao SV, McCoy LA, Spertus JA, Krone RJ, Singh M, Fitzgerald S, et al An updated bleeding model to predict the risk of post-procedure bleeding among patients undergoing percutaneous coronary intervention: a report using an expanded bleeding definition from the National Cardiovascular Data Registry CathPCI Registry. JACC Cardiovasc Interv. 2013; 6:897–904. 10.1016/j.jcin.2013.04.016 24050858

[19] Ko DT, Yun L, Wijeysundera HC, Jackevicius CA, Rao SV, Austin PC, et al Incidence, Predictors, and Prognostic Implications of Hospitalization for Late Bleeding After Percutaneous Coronary Intervention for Patients Older Than 65 Years. Circ Cardiovasc Interv. 2010; 3:140–147. 10.1161/CIRCINTERVENTIONS.109.928721 20332382

[20] Secemsky EA, Yeh RW, Kereiakes DJ, Cutlip DE, Cohen DJ, Steg PG, et al Mortality Following Cardiovascular and Bleeding Events Occurring Beyond 1 Year After Coronary Stenting: A Secondary Analysis of the Dual Antiplatelet Therapy (DAPT) Study. JAMA. Cardiol 2017;2:478–487. 10.1001/jamacardio.2017.0063 28297015 PMC5814981

[21] Mercaldi CJ, Ciarametaro M, Hahn B, Chalissery G, Reynolds MW, Sander SD, et al Cost Efficiency of Anticoagulation With Warfarin to Prevent Stroke in Medicare Beneficiaries With Nonvalvular Atrial Fibrillation. Stroke. 2011; 42:112–118. 10.1161/STROKEAHA.110.592907 21148442

[22] Deitelzweig S, Bruno A, Trocio J, Tate N, Gupta K, Lin J, et al An early evaluation of bleeding-related hospital readmissions among hospitalized patients with nonvalvular atrial fibrillation treated with direct oral anticoagulants. Curr Med Res Opin. 2016; 32:573–582. 10.1185/03007995.2015.1131676 26652179

[23] Yeh RW, Secemsky E, Kereiakes DJ, Normand SL, Gershlick AH, Cohen DJ, et al Development and validation of a prediction rule for benefit and harm of dual antiplatelet therapy beyond 1 year after percutaneous coronary intervention. JAMA. 2016;315:1735–1749. 10.1001/jama.2016.3775 27022822 PMC5408574

[24] Costa F, van Klaveren D, James S, Heg D, Raber L, Feres F, et al Derivation and validation of the predicting bleeding complications in patients undergoing stent implantation and subsequent dual antiplatelet therapy (PRECISE-DAPT) score: a pooled analysis of individual-patient datasets from clinical trials. Lancet. 2017;389:1025–1034. 10.1016/S0140-6736(17)30397-5 28290994

[25] Baber U, Mehran R, Giustino G, Cohen DJ, Henry TD, Sartori S, et al Coronary thrombosis and major bleeding after PCI with drug-eluting stents: risk scores from PARIS. J Am Coll Cardiol. 2016;67:2224–2234. 10.1016/j.jacc.2016.02.064 27079334

[26] Pisters R, Lane DA, Nieuwlaat R, de Vos CB, Crijns HJ, Lip GY. A novel user-friendly score (HAS-BLED) to assess 1-year risk of major bleeding in patients with atrial fibrillation: the Euro Heart Survey. Chest. 2010; 138:1093–1100. 10.1378/chest.10-0134 20299623

[27] Dewilde WJ, Oirbans T, Verheugt FW, Kelder JC, De Smet BJ, Herrman JP, et al Use of clopidogrel with or without aspirin in patients taking oral anticoagulant therapy and undergoing percutaneous coronary intervention: an open-label, randomised, controlled trial. Lancet. 2013;381:1107–1115. 10.1016/S0140-6736(12)62177-1 23415013

[28] Gibson CM, Mehran R, Bode C, Halperin J, Verheugt FW, Wildgoose P, et al Prevention of bleeding in patients with atrial fibrillation undergoing PCI. N Engl J Med. 2016;375:2423–2434. 10.1056/NEJMoa1611594 27959713

[29] Cannon CP, Bhatt DL, Oldgren J, Lip GYH, Ellis SG, Kimura T, et al Dual Antithrombotic Therapy with Dabigatran after PCI in Atrial Fibrillation. N Engl J Med. 2017;377:1513–1524. 10.1056/NEJMoa1708454 28844193

[30] Wittkowsky AK. Impact of target-specific oral anticoagulants on transitions of care and outpatient care models. J Thromb Thrombolysis. 2013;35:304–311. 10.1007/s11239-013-0879-y 23479258

