# Supplementary figures and images for: Causes and predictors of early readmission after percutaneous coronary intervention among patients discharged on oral anticoagulant therapy
Source: PLoS One. 2018 Oct 31;13(10):e0205457. doi: 10.1371/journal.pone.0205457 (PMC6209191; doi:10.1371/journal.pone.0205457)

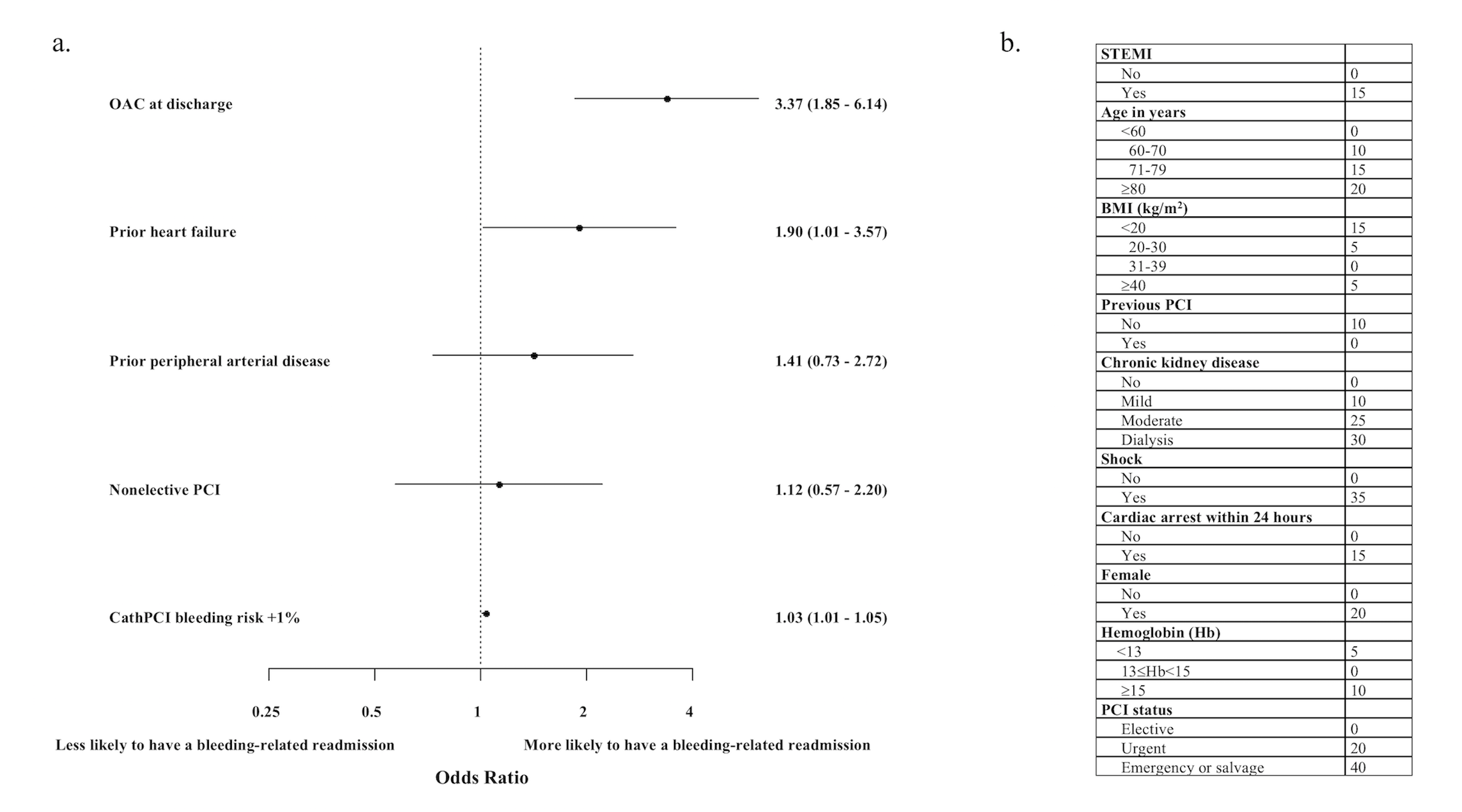

Supplement: S1 Fig — a) Predictors of bleeding-related readmissions among all patients. b) Components of the CathPCI bleeding risk score. BMI, body mass index; Hb, hemoglobin; OAC, oral anticoagulant; PCI, percutaneous coronary intervention; STEMI, ST-segment elevation myocardial infarction. (TIFF) [file pone.0205457.s001.tiff]

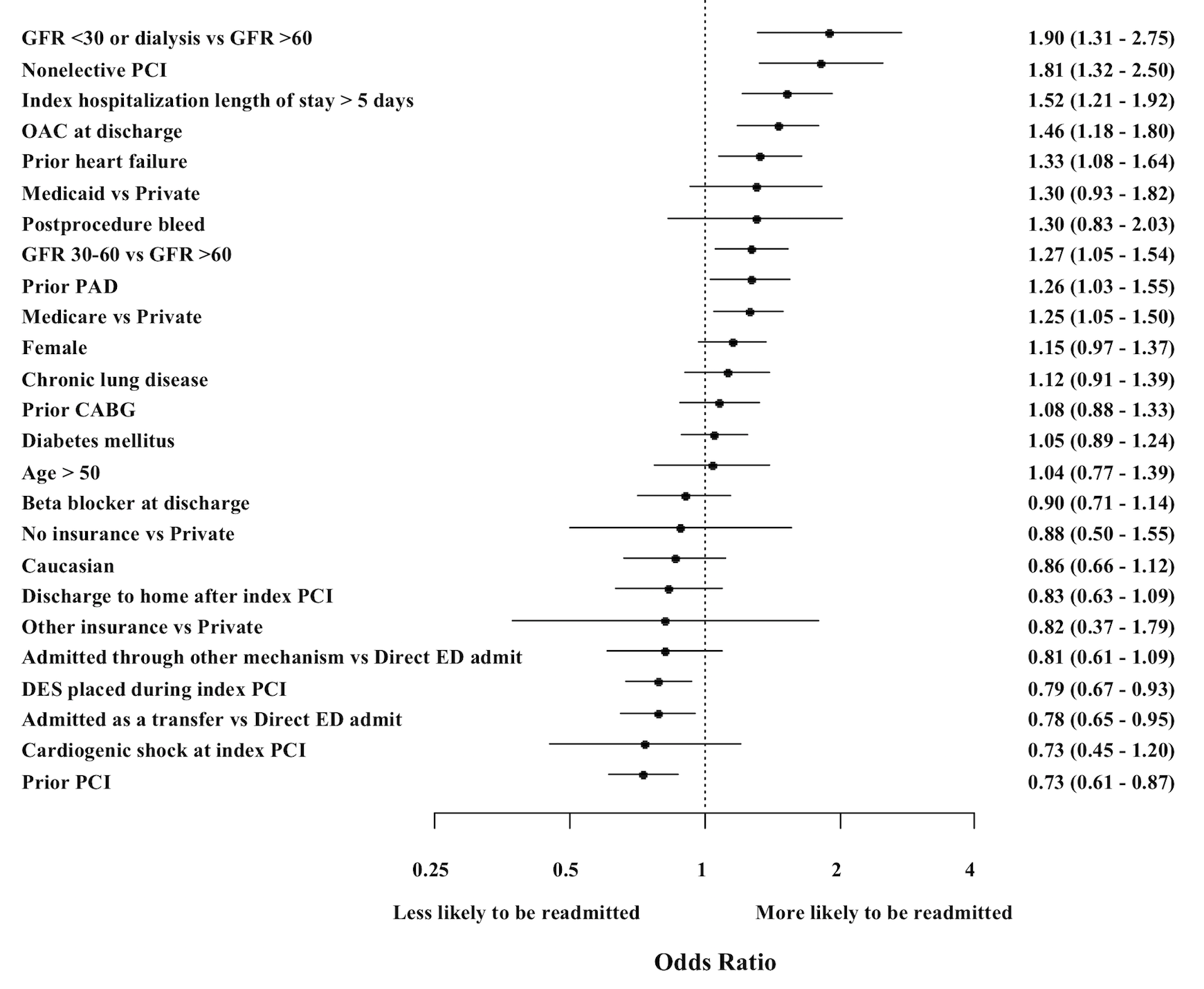

Supplement: S2 Fig — CABG, coronary artery bypass graft surgery; DES, drug eluting stent; GFR, glomerular filtration rate (mL/minute); OAC, oral anticoagulant; PAD, peripheral arterial disease; PCI, percutaneous coronary intervention. (TIFF) [file pone.0205457.s002.tiff]
