# Supplementary material for: Causes and predictors of early readmission after percutaneous coronary intervention among patients discharged on oral anticoagulant therapy
Source: PLoS One. 2018 Oct 31;13(10):e0205457. doi: 10.1371/journal.pone.0205457 (PMC6209191; doi:10.1371/journal.pone.0205457)
Supplement: S1 Table — Data are shown as n (%) except where otherwise noted. BMI, body mass index; CABG, coronary artery bypass graft surgery; CAD, coronary artery disease; CVA, cerebrovascular accident; DES, drug eluting stent; MI, myocardial infarction; NSTEMI, non-ST-segment elevation myocardial infarction; OAC, oral anticoagulant; PCI, percutaneous coronary intervention; SD, standard deviation; STEMI, ST-segment elevation myocardial infarction. (DOCX) [file pone.0205457.s003.docx]

**S1 Table. Characteristics of patients on oral anticoagulant (OAC) therapy, stratified by readmission status.**

| Characteristics | Readmitted within 30 days (n=143) | Not readmitted within 30 days (n=967) | *P* value |
| --- | --- | --- | --- |
| Age (years, mean ± SD) | 70.1 ± 12.2 | 69.5 ± 12.0 | 0.58 |
| Male | 100 (69.9) | 721 (74.6) | 0.24 |
| BMI (kg/m^2^, mean ± SD) | 29.3 ± 7.0 | 29.4 ± 6.5 | 0.84 |
| White | 132 (92.3) | 884 (91.4) | 0.72 |
| Hypertension | 124 (86.7) | 810 (83.8) | 0.37 |
| Dyslipidemia | 128 (89.5) | 893 (92.4) | 0.24 |
| Diabetes mellitus | 44 (30.8) | 373 (38.6) | 0.07 |
| Renal failure (currently on dialysis or creatinine > 2 mg/dL) | 18 (12.6) | 67 (6.9) | 0.02 |
| Current or recent smoker (within 1 year) | 20 (14.0) | 128 (13.2) | 0.81 |
| Family history of premature CAD | 21 (14.7) | 162 (16.8) | 0.53 |
| Prior MI | 56 (39.2) | 427 (44.2) | 0.26 |
| Prior PCI | 35 (24.5) | 364 (37.6) | <0.01 |
| Prior CABG | 30 (21.0) | 268 (27.7) | 0.09 |
| Prior valve surgery or procedure | 20 (14.0) | 96 (9.9) | 0.14 |
| Cerebrovascular disease | 34 (23.8) | 228 (23.6) | 0.96 |
| Prior heart failure | 44 (30.8) | 320 (33.1) | 0.58 |
| Peripheral arterial disease | 41 (28.7) | 186 (19.2) | <0.01 |
| Chronic lung disease | 29 (20.3) | 168 (17.4) | 0.40 |
| Indication for oral anticoagulation | | | |
| Nonvalvular atrial  fibrillation | 68 (47.6) | 523 (54.1) | 0.14 |
| Left ventricle  thrombus | 19 (13.3) | 105 (10.9) | 0.39 |
| Valvular atrial  fibrillation | 13 (9.1) | 80 (8.3) | 0.74 |
| Pulmonary  embolus | 12 (8.4) | 70 (7.2) | 0.62 |
| Deep vein  thrombosis | 11 (7.7) | 76 (7.9) | 0.94 |
| Left ventricle  aneurysm | 11 (7.7) | 49 (5.1) | 0.20 |
| Cardioembolic  stroke | 9 (6.3) | 35 (3.6) | 0.13 |
| Atrial flutter | 7 (4.9) | 70 (7.2) | 0.30 |
| Hypercoagulable  syndrome | 7 (4.9) | 56 (5.8) | 0.67 |
| Valvular disease | 7 (4.9) | 35 (3.6) | 0.46 |
| Other | 5 (3.5) | 44 (4.6) | 0.57 |
| Insurance | | | |
| Medicare | 85 (59.4) | 570 (59.0) | 0.91 |
| Medicaid | 8 (5.6) | 34 (3.5) | 0.22 |
| Private | 53 (37.1) | 369 (38.2) | 0.80 |
| None | 1 (0.7) | 20 (2.1) | 0.26 |
| Presentation type | | | <0.01 |
| Stable angina | 11 (7.7) | 149 (15.4) | 0.01 |
| Unstable angina | 33 (23.1) | 232 (24.0) | 0.81 |
| NSTEMI | 37 (25.9) | 214 (22.1) | 0.32 |
| STEMI | 37 (25.9) | 155 (16.0) | <0.01 |
| No symptoms / no  angina | 19 (13.3) | 192 (19.9) | 0.06 |
| Symptoms  unlikely to be  ischemic | 6 (4.2) | 25 (2.6) | 0.28 |
| PCI status | | |  |
| Elective | 23 (16.1) | 294 (30.4) | <0.01 |
| Urgent | 77 (53.9) | 476 (49.2) | 0.30 |
| Emergency | 42 (29.4) | 197 (20.4) | 0.01 |
| Salvage | 1 (0.7) | 0 (0.0) | <0.01 |
| Cardiogenic shock within 24 hours | 9 (6.3) | 35 (3.6) | 0.13 |
| DES placed | 54 (37.8) | 403 (41.7) | 0.37 |
| Post-procedure complications | | | |
| CVA | 1 (0.7) | 8 (0.8) | 0.87 |
| MI | 3 (2.1) | 27 (2.8) | 0.63 |
| Bleeding event | 23 (16.1) | 113 (11.7) | 0.13 |
| Non-access site  bleeding | 18 (78.3) | 97 (85.8) | 0.35 |
| Access site  bleeding | 5 (21.7) | 16 (14.2) | 0.13 |
| Length of stay  (days, mean ± SD) | 7.5 ± 11.1 | 4.9 ± 6.2 | <0.01 |
| OAC at discharge from index PCI |  |  | 0.30 |
| Warfarin | 138 (96.5) | 909 (94.0) | -- |
| Rivaroxaban | 3 (2.1) | 29 (3.0) | -- |
| Apixaban | 1 (0.7) | 20 (2.1) | -- |
| Dabigatran | 0 (0.0) | 3 (0.3) | -- |
| P2Y_12_ inhibitor at discharge from index PCI |  |  |  |
| Clopidogrel | 125 (87.4) | 882 (91.2) | 0.14 |
| Ticagrelor | 4 (2.8) | 24 (2.5) | 0.82 |
| Prasugrel | 1 (0.7) | 13 (1.3) | 0.52 |

Data are shown as n (%) except where otherwise noted. BMI, body mass index; CABG, coronary artery bypass graft surgery; CAD, coronary artery disease; CVA, cerebrovascular accident; DES, drug eluting stent; MI, myocardial infarction; NSTEMI, non-ST-segment elevation myocardial infarction; OAC, oral anticoagulant; PCI, percutaneous coronary intervention; SD, standard deviation; STEMI, ST-segment elevation myocardial infarction.
