# Supplementary material for: Causes and predictors of early readmission after percutaneous coronary intervention among patients discharged on oral anticoagulant therapy
Source: PLoS One. 2018 Oct 31;13(10):e0205457. doi: 10.1371/journal.pone.0205457 (PMC6209191; doi:10.1371/journal.pone.0205457)
Supplement: S2 Table — Data are shown as n (%) except where otherwise noted. CABG, coronary artery bypass graft surgery; CRT, cardiac resynchronization therapy; ICD, implantable cardioverter-defibrillator; MSK, musculoskeletal; NSTEMI, non-ST-segment elevation myocardial infarction; OAC, oral anticoagulant; PCI, percutaneous coronary intervention; STEMI, ST-segment elevation myocardial infarction; TIA, transient ischemic attack; URI, upper respiratory tract infection. (DOCX) [file pone.0205457.s004.docx]

**S2 Table. Complete list of reasons for readmission.**

| Reasons | OAC at Discharge (n=143) | No OAC at Discharge  (n=549) |
| --- | --- | --- |
| Chest pain syndromes | 31 (21.7) | 189 (34.4) |
| Stable angina | 3 (9.7) | 21 (11.1) |
| Unstable angina | 10 (32.3) | 63 (33.3) |
| NSTEMI | 2 (6.5) | 32 (16.9) |
| STEMI | 1 (3.2) | 0 (0.0) |
| Noncardiac chest pain | 10 (32.3) | 67 (35.4) |
| Pericarditis | 4 (12.9) | 5 (2.7) |
| Bleeding | 20 (14.0) | 33 (6.0) |
| Gastrointestinal | 17 (85.0) | 23 (69.7) |
| Access site | 1 (5.0) | 2 (6.1) |
| Genitourinary | 0 (0.0) | 3 (9.1) |
| Intracranial | 0 (0.0) | 1 (3.0) |
| Other | 2 (10.0) | 4 (12.1) |
| Epistaxis | 1 (5.0) | 0 (0.0) |
| Skin/MSK | 1 (5.0) | 2 (6.1) |
| Pulmonary | 0 (0.0) | 2 (6.1) |
| Congestive heart failure | 19 (13.3) | 53 (9.7) |
| Elective peripheral procedure or surgery | 6 (4.2) | 16 (2.9) |
| Stroke or TIA (not related to PCI) | 6 (4.2) | 9 (1.6) |
| Atrial fibrillation | 5 (3.5) | 9 (1.6) |
| Syncope or presyncope | 5 (3.5) | 26 (4.7) |
| Aortic stenosis | 4 (2.8) | 7 (1.3) |
| Stent thrombosis | 4 (2.8) | 14 (2.6) |
| Pneumonia | 3 (2.1) | 8 (1.5) |
| Vascular complication of PCI (aneurysm, fistula) | 3 (2.1) | 4 (0.7) |
| Venous thromboembolism | 3 (2.1) | 3 (0.6) |
| Ventricular tachycardia | 3 (2.1) | 6 (1.1) |
| Bradycardia | 2 (1.4) | 1 (0.2) |
| Elective CABG | 2 (1.4) | 11 (2.0) |
| Hypotension | 2 (1.4) | 5 (0.9) |
| Bacteremia or endocarditis | 1 (0.7) | 4 (0.7) |
| Cholecystitis, gastroenteritis, colitis/enteritis, pancreatitis, cholangitis, or abdominal pain | 1 (0.7) | 23 (4.2) |
| Elective ICD/CRT placement | 1 (0.7) | 3 (0.6) |
| Sepsis | 1 (0.7) | 7 (1.3) |
| Staged PCI without new symptoms | 1 (0.7) | 10 (1.8) |
| Viral infection, URI, bronchitis | 1 (0.7) | 3 (0.6) |
| Anxiety, depression, or panic attack | 0 (0.0) | 2 (0.4) |
| Chronic obstructive pulmonary disease | 0 (0.0) | 3 (0.6) |
| Fever | 0 (0.0) | 4 (0.7) |
| Renal failure | 0 (0.0) | 9 (1.6) |
| Rhabdomyolysis | 0 (0.0) | 2 (0.4) |
| Urinary tract infection or urosepsis | 0 (0.0) | 8 (1.5) |
| Skin and soft tissue infection | 2 (1.4) | 6 (1.1) |
| Allergic reaction | 0 (0.0) | 4 (0.7) |
| Gastroesophageal reflux disease | 0 (0.0) | 4 (0.7) |
| Bowel obstruction | 0 (0.0) | 3 (0.6) |
| Fall | 1 (0.7) | 2 (0.4) |
| Fatigue | 0 (0.0) | 3 (0.6) |
| Hypertension | 0 (0.0) | 4 (0.7) |
| Limb ischemia | 1 (0.7) | 2 (0.4) |
| Diarrhea | 1 (0.7) | 2 (0.4) |
| Elective pacemaker placement or revision | 0 (0.0) | 2 (0.4) |
| Musculoskeletal pain | 1 (0.7) | 1 (0.2) |
| Nausea/vomiting | 0 (0.0) | 2 (0.4) |
| Osteomyelitis | 1 (0.7) | 1 (0.2) |
| Seizure | 0 (0.0) | 2 (0.4) |
| Supraventricular tachycardia | 1 (0.7) | 1 (0.2) |
| Arteriovenous fistula thrombosis | 0 (0.0) | 1 (0.2) |
| Altered mental status | 0 (0.0) | 2 (0.4) |
| Anemia of unclear etiology | 0 (0.0) | 1 (0.2) |
| Cardiac tamponade or pericardial effusion | 1 (0.7) | 1 (0.2) |
| Cord compression | 0 (0.0) | 1 (0.2) |
| Dental infection | 0 (0.0) | 1 (0.2) |
| Flushing | 0 (0.0) | 1 (0.2) |
| Hyperkalemia | 0 (0.0) | 2 (0.4) |
| Hypoglycemia | 0 (0.0) | 1 (0.2) |
| Hyponatremia | 0 (0.0) | 1 (0.2) |
| Interstitial lung disease | 0 (0.0) | 1 (0.2) |
| Jejunostomy tube leakage | 0 (0.0) | 1 (0.2) |
| Joint replacement | 1 (0.7) | 0 (0.0) |
| Valve repair/replacement | 2 (1.4) | 1 (0.2) |
| Opioid withdrawal | 0 (0.0) | 1 (0.2) |
| Pernicious anemia | 1 (0.7) | 0 (0.0) |
| Reactive airways disease | 0 (0.0) | 1 (0.2) |
| Shortness of breath due to deconditioning | 1 (0.7) | 1 (0.2) |
| Temporomandibular joint disorder or migraine variant | 0 (0.0) | 1 (0.2) |
| Abdominal aortic aneurysm repair | 0 (0.0) | 1 (0.2) |
| Cardiac arrest | 0 (0.0) | 1 (0.2) |
| Cardiac monitoring during chemotherapy | 0 (0.0) | 1 (0.2) |
| Diplopia | 0 (0.0) | 1 (0.2) |
| Dysphagia | 0 (0.0) | 1 (0.2) |
| Elective neurostimulator placement | 0 (0.0) | 1 (0.2) |
| Malignant ascites | 0 (0.0) | 1 (0.2) |
| Pancreatic adenocarcinoma | 0 (0.0) | 1 (0.2) |
| Peritonitis | 0 (0.0) | 1 (0.2) |
| Pleural effusion | 0 (0.0) | 2 (0.4) |
| Stasis dermatitis | 0 (0.0) | 1 (0.2) |
| Suprapubic catheter pain | 0 (0.0) | 1 (0.2) |
| Thymoma | 0 (0.0) | 1 (0.2) |
| Thyrotoxicosis | 1 (0.7) | 0 (0.0) |
| Vertigo | 0 (0.0) | 1 (0.2) |

Data are shown as n (%) except where otherwise noted. CABG, coronary artery bypass graft surgery; CRT, cardiac resynchronization therapy; ICD, implantable cardioverter-defibrillator; MSK, musculoskeletal; NSTEMI, non-ST-segment elevation myocardial infarction; OAC, oral anticoagulant; PCI, percutaneous coronary intervention; STEMI, ST-segment elevation myocardial infarction; TIA, transient ischemic attack; URI, upper respiratory tract infection.
