# Supplementary material for: Causes and predictors of early readmission after percutaneous coronary intervention among patients discharged on oral anticoagulant therapy
Source: PLoS One. 2018 Oct 31;13(10):e0205457. doi: 10.1371/journal.pone.0205457 (PMC6209191; doi:10.1371/journal.pone.0205457)
Supplement: S3 Table — Data are shown as n (%). BARC, Bleeding Academic Research Consortium; OAC, oral anticoagulant. (DOCX) [file pone.0205457.s005.docx]

**S3 Table. BARC classification of all bleeding events in readmitted patients.**

| BARC classification | OAC at discharge (n=20) | No OAC at discharge (n=33) |
| --- | --- | --- |
| 2 | 5 (25.0%) | 10 (30.3%) |
| 3a | 11 (55.0%) | 17 (51.5%) |
| 3b | 4 (20.0%) | 4 (12.1%) |
| 3c | 0 (0.0%) | 2 (6.1%) |

Data are shown as n (%). BARC, Bleeding Academic Research Consortium; OAC, oral anticoagulant.
