# Supplementary material for: Causes and predictors of early readmission after percutaneous coronary intervention among patients discharged on oral anticoagulant therapy
Source: PLoS One. 2018 Oct 31;13(10):e0205457. doi: 10.1371/journal.pone.0205457 (PMC6209191; doi:10.1371/journal.pone.0205457)
Supplement: S4 Table — Data are shown as n (%) except where otherwise noted. CABG, coronary artery bypass graft surgery; CRT, cardiac resynchronization therapy; ICD, implantable cardioverter-defibrillator; MSK, musculoskeletal; NSTEMI, non-ST-segment elevation myocardial infarction; OAC, oral anticoagulant; PCI, percutaneous coronary intervention; STEMI, ST-segment elevation myocardial infarction; TIA, transient ischemic attack; URI, upper respiratory tract infection. (DOCX) [file pone.0205457.s006.docx]

**S4 Table. Outcomes of patients on OACs during readmission, stratified by chronicity of OAC use.**

| Outcome | OAC use 30 days prior to index PCI (n=72) | No OAC use 30 days prior to index PCI  (n=71) | *P* Value |
| --- | --- | --- | --- |
| **Reasons for Readmission** | | | |
| Chest pain syndromes | 19 (26.4) | 12 (16.9) | 0.17 |
| Stable angina | 0 (0.0) | 1 (8.3) | --- |
| Unstable angina | 8 (42.1) | 2 (16.7) | --- |
| NSTEMI | 1 (5.3) | 1 (8.3) | --- |
| STEMI | 2 (10.5) | 2 (16.7) | --- |
| Noncardiac chest  pain | 6 (31.6) | 4 (33.3) | --- |
| Pericarditis | 1 (5.3) | 0 (0.0) | --- |
| Bleeding | 11 (15.3) | 9 (12.7) | 0.65 |
| Gastrointestinal | 10 (90.1) | 7 (77.8) | 0.45 |
| Access site | 1 (9.1) | 0 (0.0) | 0.32 |
| Other | 0 (0.0) | 2 (22.2) | 0.15 |
| Epistaxis | 0 (0.0) | 1 (50.0) | --- |
| Skin/MSK | 0 (0.0) | 1 (50.0) | --- |
| Congestive heart failure | 9 (12.5) | 10 (14.1) | 0.78 |
| Elective peripheral procedure or surgery | 5 (6.9) | 1 (1.4) | 0.10 |
| Stroke or TIA (not related to PCI) | 1 (1.4) | 5 (7.0) | 0.10 |
| Atrial fibrillation | 2 (2.8) | 3 (4.2) | 0.64 |
| Syncope or presyncope | 2 (2.8) | 3 (4.2) | 0.64 |
| Aortic stenosis | 4 (5.6) | 0 (0.0) | 0.04 |
| Stent thrombosis | 2 (2.8) | 2 (2.8) | 0.99 |
| Pneumonia | 0 (0.0) | 3 (4.2) | 0.08 |
| Vascular complication of PCI (aneurysm, fistula) | 3 (4.2) | 0 (0.0) | 0.08 |
| Venous thromboembolism | 0 (0.0) | 3 (4.2) | 0.08 |
| Ventricular tachycardia | 2 (2.8) | 1 (1.4) | 0.57 |
| Bradycardia | 1 (1.4) | 1 (1.4) | 0.99 |
| Elective CABG | 1 (1.4) | 1 (1.4) | 0.99 |
| Hypotension | 0 (0.0) | 2 (2.8) | 0.15 |
| Bacteremia or endocarditis | 0 (0.0) | 1 (1.4) | 0.31 |
| Cholecystitis, gastroenteritis, colitis/enteritis, pancreatitis, cholangitis, or abdominal pain | 0 (0.0) | 1 (1.4) | 0.31 |
| Elective ICD/CRT placement | 1 (1.4) | 0 (0.0) | 0.31 |
| Sepsis | 0 (0.0) | 1 (1.4) | 0.31 |
| Staged PCI without new symptoms | 1 (1.4) | 0 (0.0) | 0.31 |
| Viral infection, URI, bronchitis | 0 (0.0) | 1 (1.4) | 0.31 |
| Other | 7 (9.7) | 9 (12.7) | 0.58 |
| **Died during readmission** | 4 (5.6) | 5 (7.0) | 0.71 |

Data are shown as n (%) except where otherwise noted. CABG, coronary artery bypass graft surgery; CRT, cardiac resynchronization therapy; ICD, implantable cardioverter-defibrillator; MSK, musculoskeletal; NSTEMI, non-ST-segment elevation myocardial infarction; OAC, oral anticoagulant; PCI, percutaneous coronary intervention; STEMI, ST-segment elevation myocardial infarction; TIA, transient ischemic attack; URI, upper respiratory tract infection.
