# Supplementary material for: Causes and predictors of early readmission after percutaneous coronary intervention among patients discharged on oral anticoagulant therapy
Source: PLoS One. 2018 Oct 31;13(10):e0205457. doi: 10.1371/journal.pone.0205457 (PMC6209191; doi:10.1371/journal.pone.0205457)
Supplement: S5 Table — Data are shown as n (%). ASA, aspirin; OAC, oral anticoagulant. (DOCX) [file pone.0205457.s007.docx]

**S5 Table. Medication regimens for patients discharged on oral anticoagulant therapy and readmitted.**

| **Discharge from index hospitalization** (n=143) | |
| --- | --- |
| OAC | 143 (100.0%) |
| Warfarin | 138 (96.5%) |
| Dabigatran | 0 (0.0%) |
| Rivaroxaban | 3 (2.1%) |
| Apixaban | 1 (0.7%) |
| Other | 1 (0.7%) |
| Triple therapy (OAC + ASA + P2Y_12_ inhibitor) | 130 (90.9%) |
| OAC + P2Y_12_ inhibitor | 4 (2.8%) |
| OAC + ASA | 9 (6.3%) |
| **Discharge from readmission** (n=134) | |
| OAC | 101 (75.4%) |
| Warfarin | 98 (97.0%) |
| Dabigatran | 0 (0.0%) |
| Rivaroxaban | 1 (1.0%) |
| Apixaban | 2 (2.0%) |
| Triple therapy (OAC + ASA + P2Y_12_ inhibitor) | 84 (62.7%) |
| OAC + P2Y_12_ inhibitor | 3 (2.2%) |
| OAC + ASA | 12 (9.0%) |
| OAC alone | 2 (1.5%) |
| ASA + P2Y_12_ inhibitor | 26 (19.4%) |
| ASA alone | 4 (3.0%) |
| P2Y_12_ inhibitor alone | 1 (0.8%) |
| No antiplatelet therapy or OAC | 2 (1.5%) |

Data are shown as n (%). ASA, aspirin; OAC, oral anticoagulant.
